# Though Active on RINm5F Insulinoma Cells and Cultured Pancreatic Islets, Recombinant IL-22 Fails to Modulate Cytotoxicity and Disease in a Protocol of Streptozotocin-Induced Experimental Diabetes

**DOI:** 10.3389/fphar.2015.00317

**Published:** 2016-01-12

**Authors:** Anika Berner, Malte Bachmann, Christine Bender, Josef Pfeilschifter, Urs Christen, Heiko Mühl

**Affiliations:** Pharmazentrum Frankfurt/ZAFES, University Hospital Goethe-University FrankfurtFrankfurt am Main, Germany

**Keywords:** streptozotocin-diabetes, RINm5F cells, tissue regeneration, tissue protection, STAT3 transcription factor, IL-22

## Abstract

Interleukin (IL)-22 is a cytokine displaying tissue protective and pro-regenerative functions in various preclinical disease models. Anti-bacterial, pro-proliferative, and anti-apoptotic properties mediated by activation of the transcription factor signal transducer and activator of transcription (STAT)-3 are key to biological functions of this IL-10 family member. Herein, we introduce RINm5F insulinoma cells as rat β-cell line that, under the influence of IL-22, displays activation of STAT3 with induction of its downstream gene targets *Socs3*, *Bcl3*, and *Reg3b*. In addition, IL-22 also activates STAT1 in this cell type. To refine those observations, IL-22 biological activity was evaluated using *ex vivo* cultivated murine pancreatic islets. In accord with data on RINm5F cells, islet exposure to IL-22 activated STAT3 and upregulation of STAT3-inducible *Socs3, Bcl3*, and *Steap4* was evident under those conditions. As these observations supported the hypothesis that IL-22 may exert protective functions in toxic β-cell injury, application of IL-22 was investigated in murine multiple-low-dose streptozotocin (STZ)-induced diabetes. For that purpose, recombinant IL-22 was administered thrice either immediately before and at disease onset (at d4, d6, d8) or closely thereafter (at d8, d10, d12). These two IL-22-treatment periods coincide with two early peaks of β-cell injury detectable in this model. Notably, none of the two IL-22-treatment strategies affected diabetes incidence or blood glucose levels in STZ-treated mice. Moreover, pathological changes in islet morphology analyzed 28 days after disease induction were not ameliorated by IL-22 administration. Taken together, despite being active on rat RINm5F insulinoma cells and murine pancreatic islets, recombinant IL-22 fails to protect pancreatic β-cells in the tested protocols from toxic effects of STZ and thus is unable to ameliorate disease in the widely used model of STZ-induced diabetes.

## Introduction

Interleukin (IL)-22 (Dumoutier et al., 2000) is a member of the IL-10 cytokine family that, based on cell-type restricted expression of its heterodimeric IL-10R2/IL-22R1 receptor, primarily acts on non-leukocytic foremost epithelial (like-) cells. Notably, whereas IL-10R2 is ubiquitous, IL-22R1 expression adheres to aforementioned restrictions (Wolk et al., 2004). IL-22 biological activity is tightly connected to activation of signal transducer and activator of transcription (STAT)-3, albeit efficient and functionally decisive STAT1 signaling is possible in context specific manner, particularly under the influence of interferons (IFN; Bachmann et al., 2013; Mühl, 2013; Hernández et al., 2015; Lamarthée et al., 2015). In most cases, however, IL-22 biological properties mirror STAT3 actions which characteristically include strengthening of anti-apoptotic and pro-proliferative cellular processes as well as enforcing cellular stress resistance in general (Aujla and Kolls, 2009; Wolk et al., 2010; Kong et al., 2013; Dudakov et al., 2015). Accordingly, on the one hand IL-22 has been shown to promote carcinogenesis (Lim and Savan, 2014; Waidmann et al., 2014) but, on the other hand, is likewise capable to serve protective and pro-regenerative tissue functions in the context of diverse toxic insults (Mühl et al., 2013). Disease models in which IL-22 application has been shown to ameliorate tissue damage in face of toxic challenges include acute liver injury in response to acetaminophen (Scheiermann et al., 2013; Feng et al., 2014) or alcohol (Xing et al., 2011), colitis induced by dextran sulfate sodium (Cox et al., 2012), and cerulein-induced pancreatitis (Feng et al., 2012).

Streptozotocin (STZ) is a glucosamine-nitrosourea that acts by alkylating DNA and exposing cells to reactive oxygen species and nitric oxide (Szkudelski, 2001; Lenzen, 2008). Due to expression of the Glut-2 glucose transporter, STZ efficiently accumulates in rodent pancreatic β-cells resulting in cell death, a mechanism routinely deployed to induce experimental diabetes. Specifically, administration of multiple-low-doses of STZ to mice mediates initial early β-cell apoptosis followed by leukocytic infiltration into the pancreas amplifying pathogenesis with subsequent development of diabetic symptoms (O’Brien et al., 1996; Cardinal et al., 2001; Barlow et al., 2004; Fukuda et al., 2008).

Herein, we set out to investigate effects of IL-22 on rodent pancreatic β-cells. To assess tissue protective properties of IL-22 concerning β-cell survival and stress robustness *in vivo*, recombinant cytokine was administered during the critical initiation phase of multiple-low-dose STZ-induced diabetes.

## Materials and Methods

### Reagents

Recombinant murine IL-22 was purchased from Immunotools (Friesoythe, Germany). Rat IL-22 was from R&D Systems (Frankfurt, Germany). Rat IL-6 was from Peprotech Inc. (Hamburg, Germany). Staurosporine and STZ were from Sigma-Aldrich (Taufkirchen, Germany).

### Cultivation of RINm5f Cells and Primary Murine Islets

RINm5F Insulinoma cells (ATCC, Wesel, Germany) were maintained in RPMI 1640 supplemented with 100 U/ml penicillin, 100 μg/ml streptomycin (Life Technologies, Darmstadt, Germany), and 10% heat-inactivated FCS (Biochrom, Berlin, Germany) at 37°C and 5% CO_2_. For experiments, cells were seeded on 6-well and 96-well polystyrene plates (Greiner, Frickenhausen, Germany) in aforementioned culture medium.

Murine islets of Langerhans were isolated from wildtype C57Bl/6J mice and cultured at 37°C and 5% CO_2_ in RPMI 1640 supplemented with 100 U/ml penicillin, 100 μg/ml streptomycin, 1% non-essential amino acids, 1% pyruvate (Life Technologies), and 10% heat-inactivated FCS (Biochrom). Briefly, the pancreas was dissected and perfused with collagenase P solution (1.2 U/ml in RPMI 1640, Roche Applied Science, Mannheim, Germany). Islets were isolated by density gradient centrifugation using Ficoll Paque Plus (GE Healthcare, München, Germany), were washed, and handpicked thereafter. Isolation by combined density gradient centrifugation and subsequent handpicking as performed herein results in highly pure cultures with islet purity >99% (Ramírez-Domínguez and Castaño, 2015). After 48–96 h recovery in aforementioned medium, 60–100 islets (as specified in the respective figure legends) were seeded on 12-well polystyrene plates (Greiner, Frickenhausen).

### Detection Of *Il22r1* And *Il10r2* mRNA by Standard Polymerase Chain Reaction (PCR)

Total RNA, isolated by Tri-Reagent (Sigma–Aldrich, Taufkirchen, Germany) or NucleoSpin RNA XS columns (RNA from islets, Macherey and Nagel, Düren, Germany) was transcribed using random hexameric primers and Moloney virus reverse transcriptase (Life Technologies). The following sequence was performed for each PCR reaction: IL-22R1 and IL-10R2, 95°C for 10 min (1 cycle); followed by 95°C for 30 s, 60°C for 30s, and 72°C for 1 min (39 cycles); followed by a final extension phase at 72°C for 7 min. The following primers were used: mouse IL-22R1, forward: 5′-GTGGAATATAAGAAATACGGAGAGAG-3′ and reverse: 5′-TTCAAGGTGCATCTGGTAGGTG-3′; mouse IL-10R2, forward: 5′-GGGACTAATGAGAAGTTTCAAGTTG-3′ and reverse: 5′-CCAGAAACTCCTTCAGGTGC-3′; rat IL22R1, forward: 5′-TGCCCGATCGGACGTGG-3′ and reverse: 5′-GGCTGCACCTCAGGGAG-3′; rat IL10R2, forward: 5′-TGGAAGACACCATTATCGGACC-3′ and reverse: 5′-GGGAGGGGTTGTTTCATCAC-3′. The possibility of amplification of contaminating genomic DNA was eliminated by selecting amplicons that cross exon/intron boundaries. PCR products (murine IL-22R1, 379 bp; murine IL-10R2, 268 bp; rat IL-22R1, 420 bp; rat IL-10R2, 337 bp) were run on a 1.5% agarose gel containing 0.5 μg/ml ethidium bromide.

### Detection of *Socs3, Bcl3, Steap4*, and *Reg3b* mRNA by Realtime PCR

During realtime PCR, changes in fluorescence are caused by the Taq polymerase degrading the probe that contains a fluorescent dye [VIC, glycerinaldehyd-3-phosphate-dehydrogenase (GAPDH); all others: FAM; Life Technologies]. Pre-developed assay reagents (Life Technologies) were used for target gene analysis: *Bcl3 Rattus norvegicus* Rn01457652_m1, *Gapdh R. norvegicus* 4352338E, *Reg3b R. norvegicus* Rn00583920_m1, *Socs3 R. norvegicus* Rn00585674_s1, *Bcl3 Mus musculus* Mm00504306_m1, *Gapdh M. musculus* 4352339E, *Steap4 M. musculus* Mm00475405_m1, *Socs3 M. musculus* Mm00545913_s1. Assay-mix was from Life Technologies. Realtime PCR was performed on AbiPrism7500 Fast Sequence Detector (Life Technologies): two initial steps at 50°C for 2 min and at 95°C for 20 s were followed by 40 cycles at 95°C for 3 s and 60°C for 30s. Detection of the dequenched probe, calculation of threshold cycles (Ct values), and data analysis were performed by the Sequence Detector software. Relative changes in mRNA expression compared to unstimulated control and normalized to *Gapdh* were quantified by the 2^-ddCt^ method.

### Detection of Stat1 and Stat3 by Westernblot Analysis

Whole cell lysates were generated from RINm5F cell and islet cultures (Bachmann et al., 2007) as well as from liver tissue (Scheiermann et al., 2013) and subjected to Westernblot analysis using protocols previously described. For detection of total STAT1 or total STAT3, blots were stripped and reprobed. Antibodies: pSTAT1-Y701 (reacts against rat and murine pSTAT1), rabbit polyclonal antibody (Cell Signaling, Frankfurt, Germany, catalog #9171); pSTAT3-Y705 (reacts against rat and murine pSTAT3), rabbit polyclonal antibody (Cell Signaling, catalog #9145); total STAT1, rabbit polyclonal antibody (reacts against rat STAT1, Cell Signaling, catalog #9172); total STAT3 (reacts against rat and murine STAT3), mouse monoclonal antibody (Cell Signaling, catalog #9139).

### Cell Death Analysis

RINm5F cells (1.5 × 10^4^/well) were seeded on 96-well polystyrene plates. After 24 h of cultivation, cells were stimulated as specified. Cytotoxicity was determined based on lactate dehydrogenase release using the CytoTox 96 non-radioactive cytotoxicity assay kit (Promega, Mannheim, Germany) according to the manufacturer’s instructions.

### Transfection of RINm5f Cells and Luciferase Reporter Assays

1 μg per confluent 6-well plate of a STAT3 luciferase reporter designed to quantify the transcriptional activity of STAT3 [pGL4.47 luc2P-SIE (Promega); along with 0.5 μg pRL-CMV-Renilla (Promega)] was transiently transfected into RINm5F cells using Lipofectamine2000 (Invitrogen, Karlsruhe, Germany) according to the manufacturer’s instructions. After 4 h of transfection, medium was exchanged and cells were cultivated for further experiments as outlined in the figure legends. Thereafter, cells were harvested and luciferase activity was determined using the dual luciferase reporter assay system (Promega) and an automated chemiluminescence detector (Glomax, 96 microplate luminometer, Promega).

### The Murine Multiple-Low-Dose (Mld) Model of Stz-Induced Experimental Diabetes

Experiments using male 8-week-old C57Bl/6J mice (Charles River Laboratories, Sulzfeld, Germany) were approved by the Regierungspräsidium Darmstadt and were in accordance with NIH guidelines. Mice were sacrificed, and organs were either snap frozen in liquid nitrogen and stored at -80°C. Pancreata were immersed in Tissue-Tek OCT (Sakura Finetek, Alphen, Netherlands) and quick-frozen on dry ice.

Recombinant murine IL-22 (Immunotools, Friesoythe, Germany) dissolved in PBS was administered i.p. (3.5 μg per mouse and injection). Equal volumes of PBS served as control. To assess IL-22 bioactivity, selected mice were sacrificed 30 min after administration and liver tissue was analyzed for STAT3 activation by Westernblot analysis.

To induce diabetes, mice were injected i.p. with multiple low doses of STZ (5 mg × 55 mg STZ/kg body weight) on five consecutive days (days 1–5). STZ was dissolved in 10 mM citric acid buffer (pH 4.5) and injected within 5 min. Solvent treated mice served as controls. STZ control groups received PBS injection on days 8, 10, and 12. IL-22 was administered either on days 4, 6, and 8 (protocol-1) or on days 8, 10, and 12 (protocol-2) as outlined in the figure legends. After 28 days, mice were sacrificed.

### Determination of Blood Glucose Levels

Blood samples were obtained from the tail vein. Blood glucose was monitored with a OneTouch Ultra (LifeScan, Neckargemünd, Germany) twice weekly in unfasted mice. Animals with blood glucose values over 300 mg/dL on two consecutive days were considered diabetic.

### Immunohistochemistry

Using cryomicrotome and sialin-coated Superfrost Plus slides (Langenselbold, Germany), 7 μm tissue sections of immersed pancreata were cut. Sections were then fixed with 4% PFA and, after washing in PBS, an avidin/biotin-blocking step was included (Vector Laboratories, Peterborough, UK). Primary and biotinylated secondary antibodies (Vector Laboratories) were incubated with the sections for 60 min each. Color reaction was obtained by sequential incubation with avidin-peroxidase conjugate (Vector Laboratories) and diaminobenzidine-hydrogen peroxide (Vector Laboratories). Antibodies: polyclonal guinea pig anti-murine insulin (Dako, Glostrup, Denmark) and biotinylated rat α-guinea pig (Vector Laboratories). Sections were counterstained using hematoxylin (AppliChem, Darmstadt, Germany).

### Statistical Analysis

Data are shown as means ± SD or as means ± SEM (as indicated) and presented as fold-induction, mg/dL, or as %, [cytotoxicity, diabetes incidence, (area > 20000 mm^2^)]. For data presentation mean ± SD is chosen in case of experiments using the cell line RINm5F. In case of data involving primary cells and individual mice mean ± SEM is shown. Statistical analysis was performed as indicated in the legends by one-way ANOVA with *post hoc* Bonferroni correction (GraphPad 5.0) or unpaired Student’s *t*-test.

## Results

### Activation of Rodent Pancreatic β-Cells By Il-22 as Detected in RINm5f Insulinoma Cells

**FIGURE 1:**
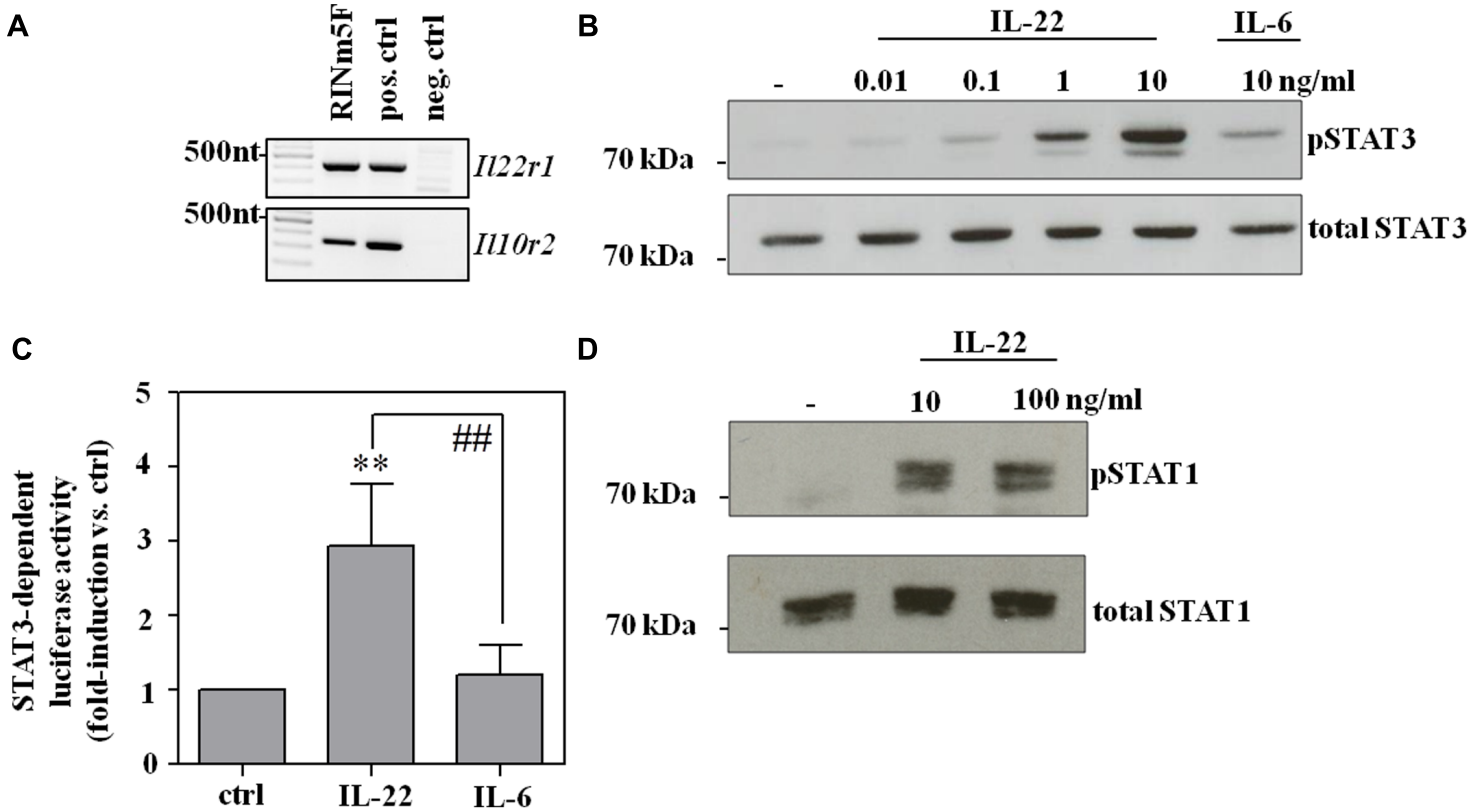
**RINm5F cells are IL-22 responsive. (A)** Untreated rat RINm5F cells were analyzed for expression of IL-22R1 and IL-10R2 mRNA by standard-PCR. cDNA obtained from rat Morris hepatoma cells and murine kidney adenocarcinoma Renca cells served as positive and negative control, respectively. Murine IL-22R1 mRNA is not detected by rat specific primers. One representative of three independently performed experiments is shown. **(B)** RINm5F cells were kept as unstimulated control, or stimulated with IL-22 (as indicated) or IL-6 (10 ng/ml) for 30 min. Whole cell lysates were analyzed for phospho-STAT3 by Westernblot analysis. After stripping the same blots were analyzed for total STAT3. One representative of three independently performed experiments is shown. **(C)** To investigate STAT3 activity, RINm5F cells were co-transfected with SIE-luciferase reporter- and CMV-renilla-plasmids. After transfection, cells were either kept as unstimulated controls or were stimulated with IL-22 or IL-6 (both 10 ng/ml) for 3 h. Thereafter, luciferase firefly activity was determined relative to renilla. Mean relative luciferase activity is expressed as fold-induction compared to unstimulated control ± SD (*n* = 4); ^∗∗^*p* < 0.01 compared with unstimulated control, ^##^*p* < 0.01. Raw data were analyzed by one-way ANOVA with *post hoc* Bonferroni correction. **(D)** RINm5F cells were kept as unstimulated control or stimulated with IL-22 (as indicated) for 30 min. Whole cell lysates were analyzed for phospho-STAT1 by Westernblot analysis. After stripping, the same blots were analyzed for total STAT1. One representative of three independently performed experiments is shown.

Rat RINm5F insulinoma cells are an established cell culture model for pancreatic β-cells used to study effects of cytokine activation and escalating cellular stress, such as exposure to overt nitric oxide (Eizirik et al., 1991; Ankarcrona et al., 1994). Herein, we introduce this β-cell line as experimental system robustly responding on a biochemical level to activation by IL-22. Accordingly, expression of both IL-22 receptor chains, namely IL-10R2 and IL-22R1 (**Figure 1A**), as well as IL-22-induced activation of STAT3 was readily detectable in RINm5F cells. The latter observation became apparent by Westernblot analysis (**Figure 1B**) and luciferase-reporter assays (**Figure 1C**), respectively. Interestingly, activation of STAT3 by IL-6, examined in these same experiments, was only modest as compared to that by IL-22 (**Figures 1B,C**). Most cytokines initiating signaling via STAT proteins, including IL-22, are capable of activating more than one type of STAT (Mühl, 2013; Villarino et al., 2015). In fact, in addition to STAT3, striking activation of STAT1 was detectable in IL-22-activated RINm5F cells (**Figure 1D**).

**FIGURE 2:**
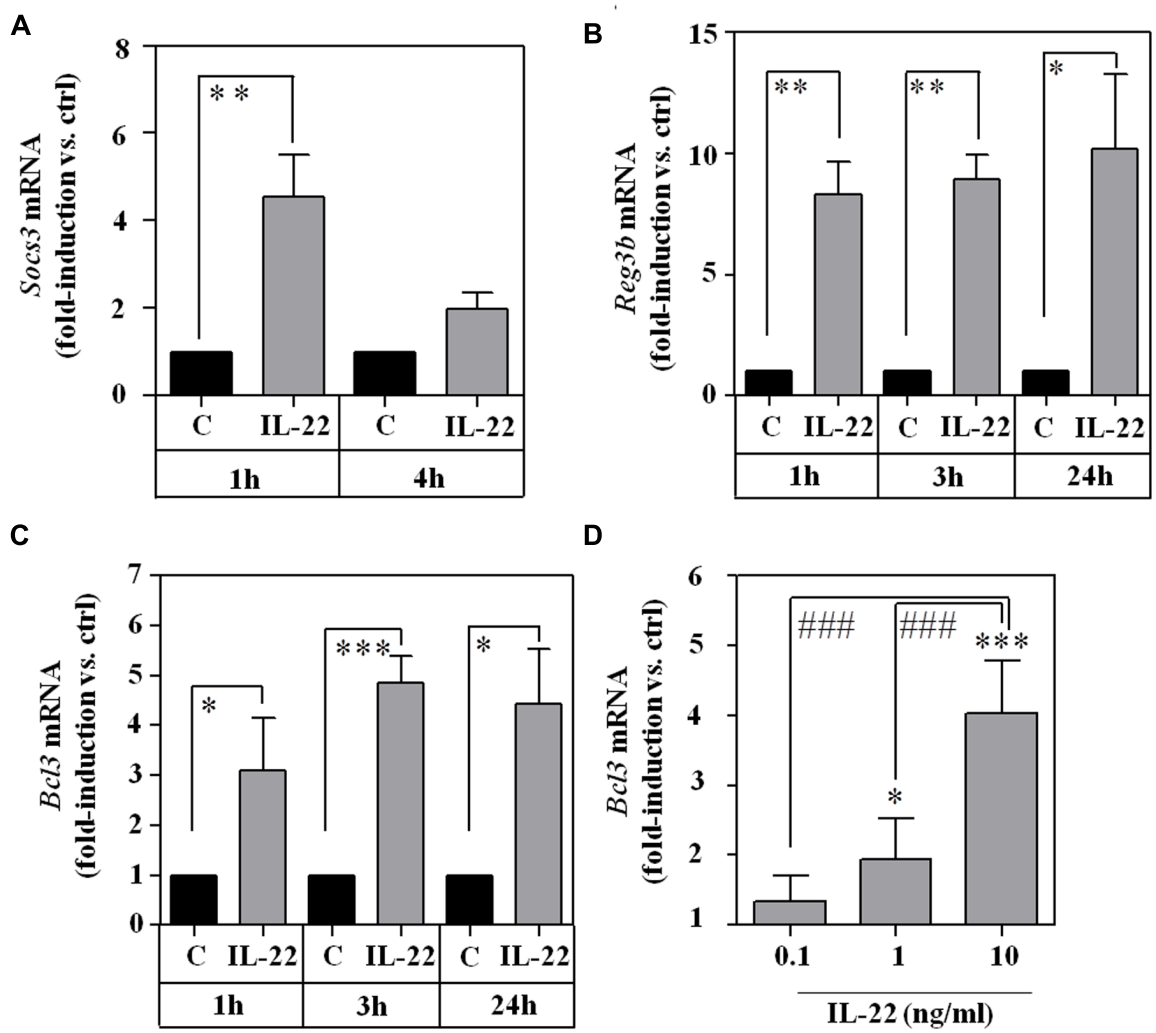
**Expression of STAT3 downstream target genes *Socs3*, *Reg3b*, and *Bcl3* in IL-22-stimulated RINm5F cells. (A–C)** RINm5F cells were kept as unstimulated control or stimulated with IL-22 (10 ng/ml) for the indicated time periods. Thereafter, target mRNA expression was determined by realtime PCR. Socs3 (*n* = 4; **A**), Reg3β (*n* = 4; **B**), and Bcl3 (*n* = 3; **C**) mRNA was normalized to that of Gapdh and is shown as mean fold-induction compared to unstimulated control ± SD; ^∗^*p* < 0.05, ^∗∗^*p* < 0.01, ^∗∗∗^*p* < 0.001. Raw data were analyzed by unpaired Student’s *t*-test. **(D)** RINm5F cells were kept as unstimulated control or stimulated with IL-22 (as indicated). After 3 h, Bcl3 mRNA expression was determined by realtime-PCR. Bcl3 mRNA was normalized to that of Gapdh and is shown as mean fold-induction compared to unstimulated control ± SD (*n* = 3); ^∗^*p* < 0.05, ^∗∗∗^*P* < 0.001 compared with unstimulated control, ^###^*p* < 0.001. Raw data were analyzed by one-way ANOVA with *post hoc* Bonferroni correction.

To assess functional consequences of RINm5F cell exposure to IL-22, prototypic STAT3-inducible genes were investigated, namely suppressor of cytokine signaling (Socs)-3 (Hoegl et al., 2011), B cell lymphoma (Bcl)-3 (Brocke-Heidrich et al., 2006), and regenerating islet-derived protein (Reg)-3β (Zheng et al., 2008; Willson et al., 2013). As shown in **Figure 2**, mRNA expression of SOCS3 (A), Reg3β (B), and Bcl3 (CD) were significantly upregulated by IL-22 as sole stimulus. Data thus clearly indicate functionality of the IL-22/STAT3 axis in RINm5F cells.

### Activation of *Ex Vivo* Cultivated Murine Primary Islets by Il-22

**FIGURE 3:**
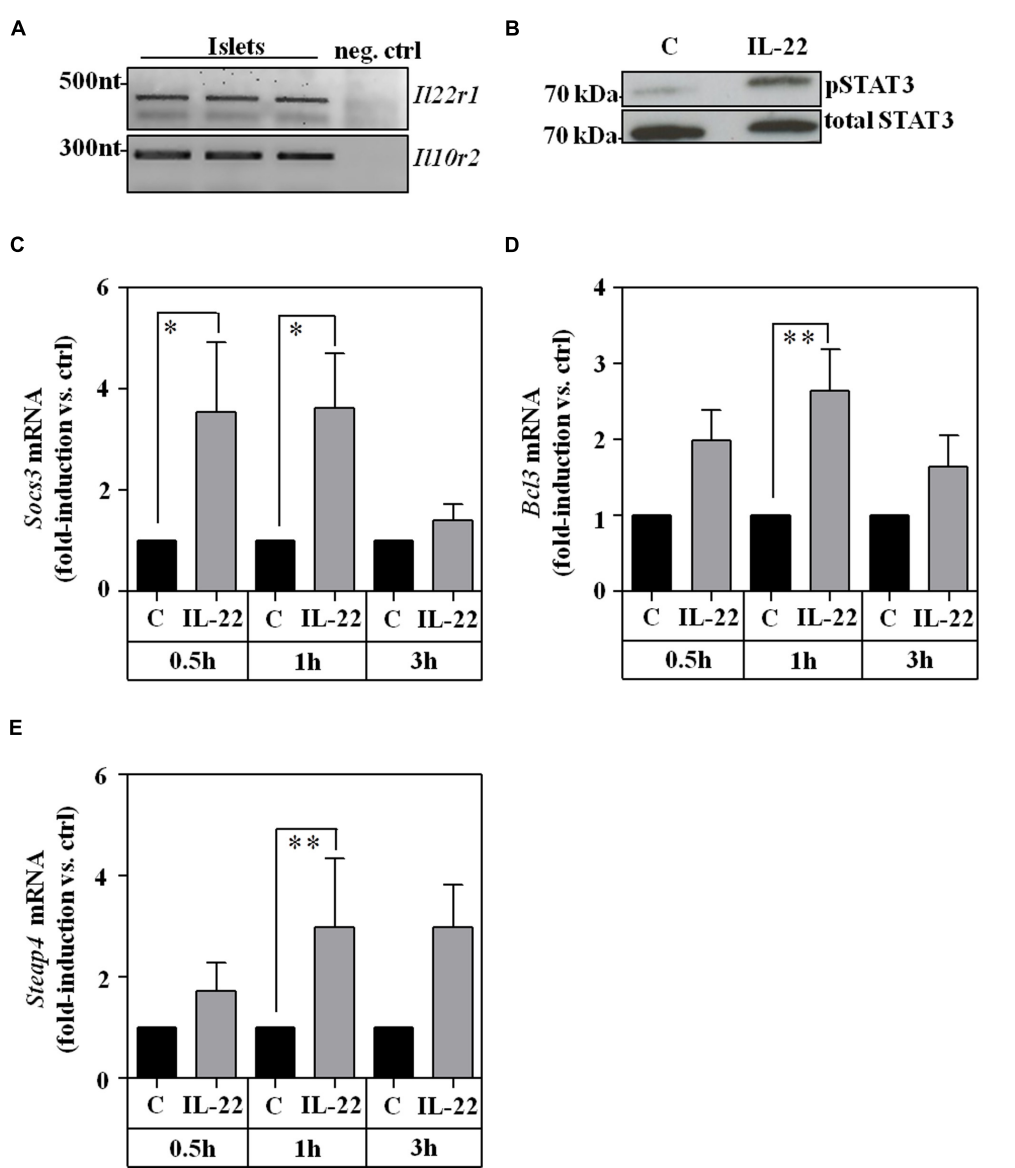
***Ex vivo* cultivated murine islets are IL-22 responsive. (A)** Islet expression of murine IL22R1 and IL10R2 mRNA was analyzed by standard PCR. One representative of three independently performed experiments is shown. cDNA from rat RINm5F cells served as negative control (neg. ctrl). Rat IL-22R1 mRNA is not detected by murine specific primers. One representative of three independently performed experiments is shown. **(B)** Islets were kept as unstimulated controls or stimulated with IL-22 (100 ng/ml). After 30 min, whole cell lysates were analyzed for phospho-STAT3 by Westernblot analysis. After stripping the same blots were analyzed for total STAT3. One representative of three independently performed experiments is shown. **(C–E)** Islets were kept as unstimulated controls or stimulated with IL-22 (20 ng/ml). After the indicated time periods, target mRNA expression was determined by realtime PCR. *Socs3* (*n* = 6; **C**), *Bcl3* (0.5 h/3 h: *n* = 6, 1 h: *n* = 8; **D**), and *Steap4* (0.5 h/3 h: *n* = 6, 1 h: *n* = 7; **E**). mRNA was normalized to that of Gapdh and is shown as mean fold-induction compared to unstimulated control ± SEM; ^∗^*p* < 0.05, ^∗∗^*p* < 0.01. Raw data were analyzed by unpaired Student’s *t*-test.

To extend those observations onto the level of primary cells, murine pancreatic islets were isolated and analyzed *ex vivo*. As with RINm5F cells, expression of IL-10R2 and IL-22R1 was readily detectable in cultured islets (**Figure 3A**) which agrees with previous proof of IL-22R1 protein on human (Shioya et al., 2008) and murine (Hasnain et al., 2014) islet β-cells. In addition, we report that *ex vivo* stimulation of murine islets with IL-22 resulted in STAT3 activation (**Figure 3B**) and upregulation of STAT3 downstream gene targets, specifically Socs3 (**Figure 3C**), Bcl3 (**Figure 3D**), and six transmembrane epithelial antigen of prostate (Steap)-4 (Ramadoss et al., 2010; **Figure 3E**). Data presented altogether confirm that murine pancreatic islets are targets of IL-22 biological activity (Hill et al., 2013; Hasnain et al., 2014).

### Administration of Il-22 Fails to Modulate Disease in a Protocol of Multiple-Low-Dose Stz-Induced Diabetes

**FIGURE 4:**
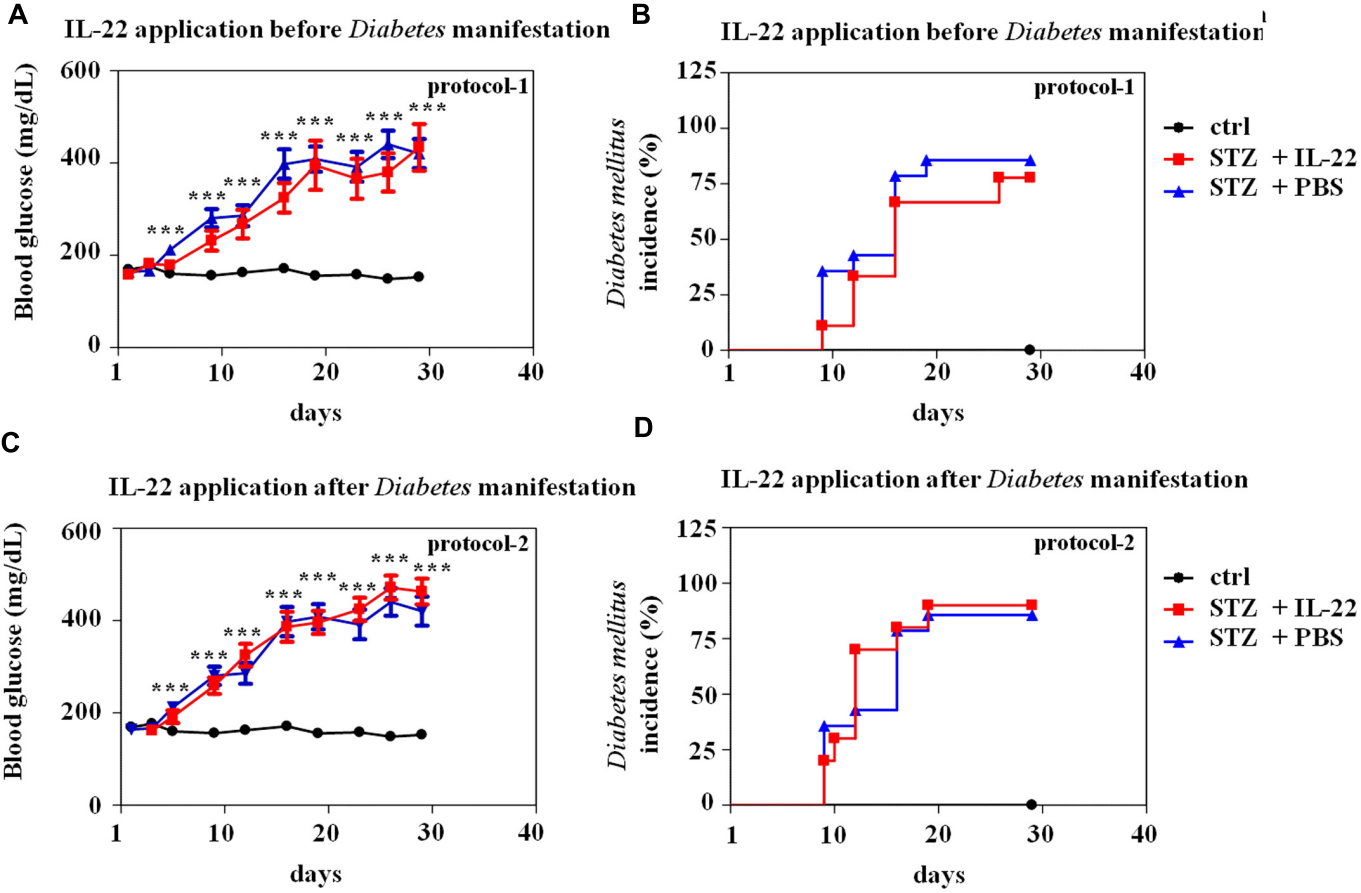
**Administration of recombinant IL-22 fails to modulate incidence and course of disease in multiple-low-dose STZ-induced diabetes.** Male C57BL/6 mice were treated with citric acid [ctrl-group black lines in **(A–D)**, *n* = 9] or five times daily with 55 mg/kg STZ (days 1–5). Those STZ-treated mice were, as indicated below, subdivided in three further groups. STZ-only [blue lines in **(A–D)**, *n* = 14] obtained for control reasons i.p. PBS on days 8, 10, and 12. STZ-protocol-1 [red lines in **(A,B)**, *n* = 9] obtained i.p. IL-22 (3.5 μg per mouse) on days 4, 6, and 8. STZ-protocol-2 [red lines in **(C,D)**, *n* = 10] obtained i.p. IL-22 (3.5 μg per mouse) on days 8, 10, and 12. All groups were monitored for 28 days. Twice weekly, blood glucose levels were analyzed from tail vein blood. Mice were defined diabetic if two consecutive blood glucose levels were ≥300 mg/dl. **(A,C)** Mean blood glucose levels are shown in mg/dl ± SEM. **(B,D)** Diabetes incidence is shown in percent. ^∗∗∗^*p* < 0.001 for STZ-only versus ctrl-group at the respective time points. Raw data were analyzed by unpaired Student’s *t*-test.

Thorough analysis of early events in the model of multiple-low-dose STZ-induced diabetes previously revealed two early peaks of β-cell apoptosis at days 5 and 11 after onset of STZ application (daily, days 1–5; O’Brien et al., 1996). Based on this former report, two regimes of treatment with recombinant IL-22 were investigated herein. First, IL-22 treatment on days 4, 6, and 8 after onset of STZ application (protocol-1). This treatment period starts days before onset of disease at around day 9 (**Figure 4**). Notably, aforementioned previous study did not detect significantly elevated rates of islet apoptosis before day 5 (O’Brien et al., 1996). The second regime consists of IL-22 treatment on days 8, 10, and 12 (protocol-2) thus starting immediately at disease onset (**Figure 4**). Both regimes involved three i.p. applications of recombinant murine IL-22 at a dosage of 3.5 μg/mouse. This same dosage was previously used in our laboratory to ameliorate acetaminophen-induced liver damage (Scheiermann et al., 2013).

**FIGURE 5:**
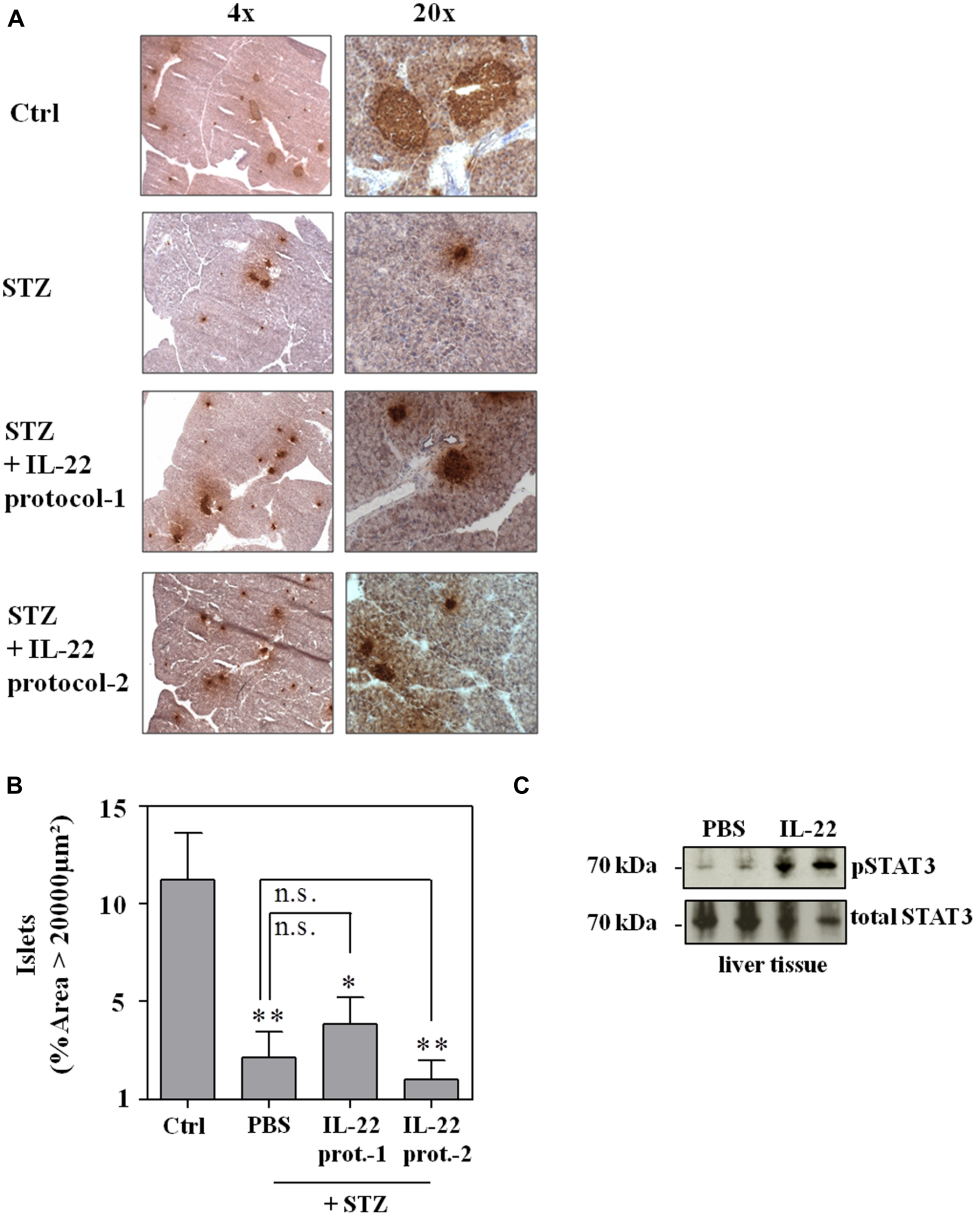
**Administration of recombinant IL-22 fails to ameliorate pancreatic morphological changes associated with multiple-low-dose STZ-induced diabetes.** Male C57BL/6 mice were treated as indicated in legend **Figure 4**. After 28 days, mice were sacrificed, pancreata harvested and imbedded for histologic analysis. **(A)** Cryosections were immunohistologically stained for insulin and counterstained with hematoxylin. Shown are representative of five analyzed pancreata per group. 4x/20x = 4-fold/ 20-fold magnification. **(B)** Immunohistological stainings of whole pancreata were analyzed in twofold magnification. All insulin positive areas per section were counted and evaluated by Image J. Shown are mean percentages of insulin positive areas ≥20000 μm^2^ ± SEM (*n* = 5); ^∗^*p* < 0.05, ^∗∗^*p* < 0.01 compared with citric acid-treated ctrl-group. Data were analyzed using one-way ANOVA with *post hoc* Bonferroni correction. n.s., not significant. **(C)** Male C57BL/6 mice were either treated with PBS or 3.5 μg IL-22. After 30 min, mice were sacrificed and organs harvested. Liver lysates were analyzed for phospho-STAT3 content by Westernblot analysis. After stripping the same blots were analyzed for total STAT3 levels. Each lane represents an individual mouse.

As shown in **Figure 4**, neither IL-22 treatment protocol-1 (AB) nor protocol-2 (CD) changed onset, course, or incidence of STZ-induced diabetes. This observation was confirmed on the morphological level by immunohistochemical analysis and quantification of insulin-positive pancreatic areas in animals under investigation. Notably, STZ-induced diabetes typically associates with a more scattered and dense appearance of insulin-positive islets (**Figure 5A**). This process can be quantified by counting the pancreatic fraction of insulin-positive islet areas larger than 20000 μm^2^ (Bellenger et al., 2011). As shown in **Figure 5B**, multiple-low-dose STZ treatment in fact mediated a significant drop in (islet area > 20000 μm^2^) which, however, was not significantly affected by IL-22 treatment protocol-1 or -2. In order to confirm the biological activity of recombinant IL-22 *in vivo*, STAT3 activation in liver tissue was determined 30 min after i.p. injection of the cytokine. In accord with previous observations (Scheiermann et al., 2013), Westernblot analysis revealed robust activation of hepatic STAT3 in response to administration of recombinant IL-22 to mice (**Figure 5C**).

**FIGURE 6:**
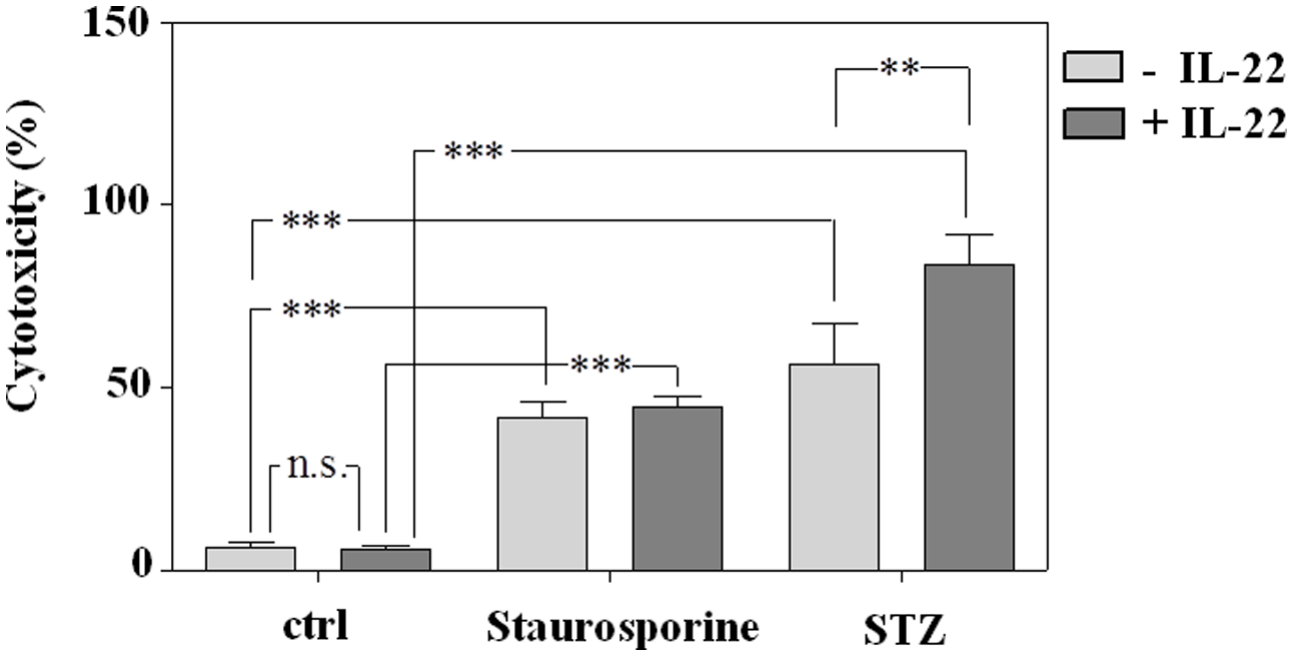
**Induction of death in RINm5F cells by exposure to staurosporine or STZ is not prevented by pre-incubation with IL-22.** RINm5F cells were kept as unstimulated control or stimulated with IL-22 (10 ng/ml). After 24 h, staurosporine (500 nM) or STZ (5 mM) were added as indicated for further 16 h. Thereafter, supernatants and cell lysates were harvested, and cytotoxicity was determined as outlined in the section “Materials and Methods.” Shown is the mean percent cytotoxicity ± SD (*n* = 3); ^∗∗^*p* < 0.01, ^∗∗∗^*p* < 0.001; n.s., not significant. Data were analyzed by one-way ANOVA with *post hoc* Bonferroni correction.

Despite lacking the Glut-2 glucose transporter and thus displaying reduced STZ sensitivity (Elsner et al., 2000), STZ is inherently able to mediate apoptotic cell death in RINm5F cells (Morgan et al., 1994; Koo et al., 2011) which in cell culture proceeds toward secondary necrosis. Since IL-22 is supposed to mediate anti-apoptosis by promoting expression of survival genes, RINm5F cells were pre-incubated with IL-22 before induction of cell death. In concurrence with aforementioned *in vivo* data, IL-22 likewise failed to protect RINm5F cells in culture from death under the influence of STZ. In fact, IL-22 actually enhanced cell death by STZ in RINm5F cells which may relate to activation of inflammatory STAT1 in this cell type (**Figure 1D**). Cells were incubated in parallel with prototypic pro-apoptotic staurosporine serving as positive control for induction of cell death in this cellular system (**Figure 6**).

## Discussion

The principle of IL-22 enforcing regenerative processes has been characterized best for the liver (Ren et al., 2010; Park et al., 2011), however, likely affects various other pathological conditions. In general, tissue protection by IL-22 is based on its capability to initiate in STAT3-dependent manner anti-apoptotic and mitogenic pathways (Mühl et al., 2013). Those connect to upregulation of survivin (Xu et al., 2015), Bcl-2 and/or Bcl-X_L,_ of cyclin D1 and/or c-Myc (Radaeva et al., 2004; Kong et al., 2012), and to activation of protein kinase B (Mitra et al., 2012) as well as ERK1/2 (Lejeune et al., 2002).

To support regenerative responses at the endocrine pancreas, specifically at the β-cell compartment, is a promising strategy for the development of novel therapeutics targeting diabetic diseases (Migliorini et al., 2014). Hitherto, it has become apparent that IL-22 ameliorates pancreatic β-cell stress and metabolic disease in murine obesity (Hasnain et al., 2014). Direct action of IL-22 on β-cells, however, has been a matter of debate. In fact, another study did not detect islet STAT3 activation by immunohistochemistry performed 2 h after administration of recombinant IL-22 to mice. In contrast, phospho-STAT3 was detectable in pancreatic acinar cells on these same tissue sections (Park et al., 2015). However, it cannot be excluded that this observation relates to late timing of analysis (2 h after IL-22 application) and reduced sensitivity of standard immunohistochemistry. Notably, albeit acinar cells express high levels of IL-22R1 (Aggarwal et al., 2001; Gurney, 2004), the IL-22 receptor is evidently detectable on β-cells by immunofluorescence confocal microscopy of murine C57BL/6 pancreatic islets (Hasnain et al., 2014).

Herein, we introduce the rat β-cell line RINm5F as biochemically and functionally IL-22 responsive. To assess the role of IL-22 for the endocrine pancreas more closely to primary cells but also biochemically standardized, experiments were performed using *ex vivo* cultivated islets. Data presented indicate functional IL-22 receptor expression by murine C57BL/6 islets. Accordingly, activation of STAT3 was readily detectable by Westernblot analysis after exposure of islet cultures to IL-22. Islet STAT3 activation coincided with induction of prototypic STAT3 downstream target genes. This observation concurs with a previous report on IL-22 responsiveness of NOD mice-derived islets. There, STAT3 activation was formally not investigated but a mitogenic pro-regenerative β-cell response was observed after *ex vivo* stimulation with IL-22 (Hill et al., 2013). Altogether, data from RINm5F cells and *ex vivo* cultivated islets (Hill et al., 2013 and data presented herein) as well as confocal immunofluorescence analysis of murine islets *in situ* (Hasnain et al., 2014) support the view that β-cells can serve as direct IL-22 target.

Activation of rat RINm5F cells or murine pancreatic islets by IL-22 resulted in upregulation of prototypic STAT3 inducible genes, among those interesting candidates displaying an immunoregulatory or tissue protective potential at the inflamed/stressed pancreas. Those include *SOCS3* (Murray, 2007) which in cell culture is β-cell protective (Karlsen et al., 2001), however, by curbing protective STAT3 signaling *in vivo*, seemingly is pathogenic as detected using a β-cell-specific knockdown during STZ-induced diabetes (Mori et al., 2007). In addition, *Bcl3* (Maldonado and Melendez-Zajgla, 2011) was a further gene identified herein as IL-22 inducible in pancreatic cells. Interestingly, this protein is capable of inhibiting several inflammatory processes that are regarded pivotal for autoimmune diabetes pathogenesis (Ruan et al., 2010). Similarly, *Reg3b* was upregulated by IL-22 in RINm5F cells. Notably, expression of Reg proteins has been related to pancreatic islet regeneration (Lu et al., 2006; Hill et al., 2013). Finally, we report for the first time on induction of *Steap4* by IL-22, a regulatory path recognized herein in murine islets. Steap4 is part of the β-cell stress response program, though its exact function in that context needs clarification (Sharma et al., 2015).

Altogether, data indicated that administration of recombinant IL-22 may be able to ameliorate via STAT3 β-cell stress/toxicity and consequently subsequent development of diabetes, a scenario to be investigated in the well-defined model of multiple-low-dose STZ application (Ahn et al., 2015). In order to test this hypothesis IL-22 treatment was intentionally confined to the initial disease phase for which early β-cell apoptosis is decisive (O’Brien et al., 1996). By applying this specific regime, effects of administered IL-22 on subsequently established disease can be excluded. Notably, IL-22 short-term treatment of mice is well tolerated and without apparent immediate side effects (Scheiermann et al., 2013). Despite these thoughtful consideration, we observed that IL-22 treatment did neither affect incidence nor course of multiple-low-dose STZ-induced diabetes. Data are in accord with a very recent report on that matter, albeit authors did not indicate details of their IL-22 treatment protocol (Park et al., 2015). *In vivo* data presented herein are furthermore supported by cell culture experiments demonstrating that RINm5F cell death by STZ is not inhibited but actually slightly increased under the influence of IL-22.

In summary, though biologically active on islet cells in culture, recombinant IL-22 failed to modulate disease initiation in multiple-low-dose STZ-induced diabetes. Data presented thus weaken the hypothesis that recombinant IL-22 can be used in focused short-term therapy to adequately support pancreatic islet protection and regeneration under conditions of overt β-cell stress.

## Author Contributions

AB performed experiments, analyzed the data, designed the study, wrote the paper, performed manuscript editing.

MB performed experiments, analyzed the data, contributed to manuscript editing.

CB analyzed the data, provided technical support.

JP analyzed the data, contributed to manuscript editing.

UC analyzed the data, provided technical support, contributed to manuscript editing.

HM analyzed the data, designed the study, wrote the paper, performed manuscript editing.

## Conflict of Interest Statement

The authors declare that the research was conducted in the absence of any commercial or financial relationships that could be construed as a potential conflict of interest.
